# Host and parasite genetics shape a link between *Trypanosoma cruzi* infection dynamics and chronic cardiomyopathy

**DOI:** 10.1111/cmi.12584

**Published:** 2016-05-25

**Authors:** Michael D. Lewis, Amanda Fortes Francisco, Martin C. Taylor, Shiromani Jayawardhana, John M. Kelly

**Affiliations:** ^1^ Department of Pathogen Molecular BiologyLondon School of Hygiene and Tropical MedicineKeppel StreetLondonWC1E 7HTUK; ^2^ Laboratory of Parasitic DiseasesNational Institute of Allergy and Infectious DiseasesBethesdaMaryland20892USA

## Abstract

Host and parasite diversity are suspected to be key factors in Chagas disease pathogenesis. Experimental investigation of underlying mechanisms is hampered by a lack of tools to detect scarce, pleiotropic infection foci. We developed sensitive imaging models to track *Trypanosoma cruzi* infection dynamics and quantify tissue‐specific parasite loads, with minimal sampling bias. We used this technology to investigate cardiomyopathy caused by highly divergent parasite strains in BALB/c, C3H/HeN and C57BL/6 mice. The gastrointestinal tract was unexpectedly found to be the primary site of chronic infection in all models. Immunosuppression induced expansion of parasite loads in the gut and was followed by widespread dissemination. These data indicate that differential immune control of *T. cruzi* occurs between tissues and shows that the large intestine and stomach provide permissive niches for active infection. The end‐point frequency of heart‐specific infections ranged from 0% in TcVI‐CLBR‐infected C57BL/6 to 88% in TcI‐JR‐infected C3H/HeN mice. Nevertheless, infection led to fibrotic cardiac pathology in all models. Heart disease severity was associated with the model‐dependent frequency of dissemination outside the gut and inferred cumulative heart‐specific parasite loads. We propose a model of cardiac pathogenesis driven by periodic trafficking of parasites into the heart, occurring at a frequency determined by host and parasite genetics.

## Introduction

Host‐pathogen interactions constitute many discrete outcomes between sites and over time, which cumulatively determine disease severity, treatment efficacy and development of immunity (Bumann, 2015). However, tissue‐specific pathogen dynamics are often poorly accounted for in experimental models, especially for chronic infections. A case in point is Chagas disease, caused by the protozoan parasite *Trypanosoma cruzi*. Human infections are life‐long and often asymptomatic. Cardiomyopathy develops in around one third of individuals and ~10% develop digestive tract megasyndromes (Rassi *et al.*, 2010; WHO, 2015), although there is high underlying variability of morbidity rates between geographical regions (Coura and Borges‐Pereira, 2012). Host and parasite diversity are assumed to underpin these diverse outcomes, but mechanistic explanations have yet to be developed. Recent data indicate that *T. cruzi* infections are more spatially and temporally dynamic than previously thought (Lewis *et al.*, 2014), raising questions about the relationship between local infection and cardiac pathogenesis. The potential for genetic factors to influence disease pathology means that the broader significance of these findings is yet to be established.

There are six major *T. cruzi* lineages (TcI‐VI), at least four of which derive from inter‐lineage hybridization events (Machado and Ayala, 2001; Westenberger *et al.*, 2005; Lewis *et al.*, 2011). Their geographical distribution maps broadly onto some epidemiological features of Chagas disease (Miles *et al.*, 2009). Most human infections in southern South America, where both cardiac and digestive forms of disease occur, are associated with lineages TcI, TcII, TcV and TcVI. In areas of northern South America and North America, where only cardiac disease is seen, TcI and occasionally TcIV are involved. Nevertheless, asymptomatic cases and heart disease of all severities occur widely, so virulence is unlikely to be a strictly lineage‐specific phenotype. From the host perspective, characteristics of immune responses that protect against acute mortality are relatively well established (Tarleton, 2007; Junqueira *et al.*, 2010). In contrast, factors that determine the progression and severity of chronic cardiomyopathy are poorly understood. Several candidate human genetic variants have been identified (Ramasawmy *et al.*, 2007; del Puerto *et al.*, 2012), but larger study sizes are considered necessary to establish their significance (Cunha‐Neto and Chevillard, 2014).

The role of the parasite as a driver of heart pathology has been controversial (Gironès and Fresno, 2003; Tarleton, 2003; Marin‐Neto *et al.*, 2007; Dutra and Gollob, 2008; Gutierrez *et al.*, 2009; Bonney and Engman, 2015). *Trypanosoma cruzi* is challenging to investigate in both clinical and experimental settings, largely because of the difficulty of detecting scarce, intracellular tissue parasites. This aspect of chronic infections and the presence of auto‐reactive T cells and antibodies in heart tissue led to an autoimmune hypothesis of pathogenesis (reviewed in Bonney and Engman, 2015). Molecular detection methods have since established that parasites do persist in symptomatic hosts as shown, for example, by the presence of parasite DNA or antigen in heart tissue (Higuchi *et al.*, 1993; Jones *et al.*, 1993; Añez *et al.*, 1999). It has also become clear that autoimmune phenomena require ongoing *T. cruzi* infection (Hyland *et al.*, 2007). Such data have been taken to support the idea that continual low‐grade parasitism of heart tissue sustains pathological inflammatory responses. However, support for this conjecture is surprisingly weak: no quantitative association between heart‐specific parasite loads and disease severity has been demonstrated, tissue sampling is strongly biased, molecular methods do not directly detect live organisms, histopathological analyses are often qualitative, and the majority of animal studies focus on acute infections, with unclear relevance for the chronic phase.

We developed a highly sensitive *T. cruzi* infection imaging model, based on the red‐shifted firefly luciferase variant *PpyRE9h* (Branchini *et al.*, 2010). We used this model to define the tissue‐specific infection dynamics of the *T. cruzi* CL Brener (CLBR) strain in BALB/c mice (Lewis *et al.*, 2014). In acute infections, parasites were pan‐tropic, but in chronically infected mice they were not detectable in the myocardium and almost entirely restricted to the GI tract. However, gut‐tropism could simply be a phenotype associated with this strain, lineage or the parasite‐mouse strain pairing. Here, we use *in vivo* imaging models for seven host‐parasite strain combinations to show that the GI tract is the main reservoir site for *T. cruzi* persistence, irrespective of parasite or host genetic differences. We furthermore identify model‐dependent patterns in the frequency of infection outside the gut niche that implicate periodic parasitism of the heart, rather than continuous persistence, as a key determinant of heart pathology.

## Results

### Chronic gastrointestinal tract persistence of diverse *T. cruzi* strains in BALB/c mice

Bioluminescent *T. cruzi* clones were selected for the highly divergent lineages TcI and TcVI. These are estimated to have last shared a common ancestor ~2 million years ago (Lewis *et al.*, 2011). Bioluminescent TcVI‐CLBR and TcI‐X10/6 were described previously (Lewis *et al.*, 2014; Taylor *et al.*, 2015). They exhibit stable, constitutive *PpyRE9h* expression and cause fulminant infections in severe combined immunodeficiency (SCID) mice. These features were also observed for the TcI‐JR bioluminescent clone, which was developed for this study (Fig. S1). Serial *in vivo* imaging allows the course of infection to be monitored with greater precision and sensitivity than blood microscopy or PCR‐based approaches (Lewis *et al.*, 2014; Francisco *et al.*, 2015). We thus aimed to apply bioluminescence imaging to investigate the dynamics of infections with these diverse strains in a series of mouse models.

**Figure 1:**
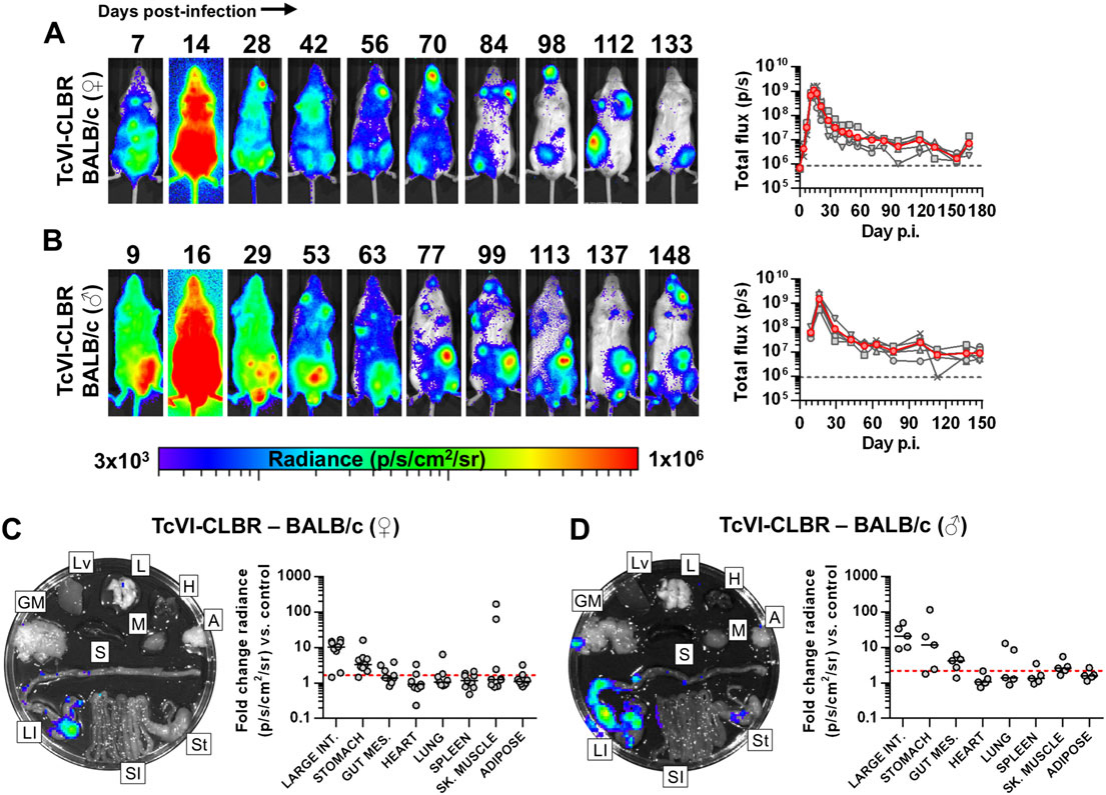
Bioluminescence imaging of *T*. *cruzi* TcVI‐CLBR in BALB/c mice reveals infection kinetics and gastrointestinal persistence. A, B. Course of TcVI‐CLBR infection in female (*n* = 10) (A) and male (*n* = 5) (B) BALB/c mice tracked by *in vivo* bioluminescence imaging. Panels show serial ventral images of one mouse. Log‐scale pseudocolour heat maps show intensity of bioluminescence; minimum and maximum radiances are indicated. Charts show total bioluminescence for five individual mice (grey) and their mean (red) from the experiment(s) represented by adjacent images. Dashed line indicates detection threshold. C, D. Quantification of tissue‐specific chronic parasite densities in female (*n* = 9, 167–168 dpi) (C) and male (*n* = 5, 151 dpi) (D) BALB/c mice using *ex vivo* bioluminescence imaging. Images show bioluminescence intensities for a representative mouse: Adipose (A), gut mesenteric tissue (GM), heart (H), lung (L), large intestine (LI), liver (Lv), skeletal muscle (M), spleen (S), small intestine (SI) and stomach (St). Charts show quantification of tissue‐specific infection intensities expressed as fold change in bioluminescence versus matching tissues from uninfected controls. Black lines show median values and red dashed lines indicate detection threshold. Data are from two independent experiments (female) and one experiment (male).

Our standard imaging model of TcVI‐CLBR infection in female BALB/c mice is sufficiently sensitive to detect as few as 100 free parasites inside the peritoneal cavity (Lewis *et al.*, 2014). This enabled serial quantification of parasite burdens for >12 months, using bioluminescence as a proxy (Lewis *et al.*, 2015). This mouse–parasite combination is characterized by a peak parasite burden at two‐weeks post‐infection (p.i.), which resolves to a chronic phase over the next 4–6 weeks. Long‐term infections persist as an approximate dynamic equilibrium, as shown by bioluminescence intensity fluctuating between 2 and 3 orders of magnitude below the acute peak (Fig. 1A). Infection foci are normally associated with the abdominal region, but also localize to highly diverse additional anatomical positions in a temporally dynamic mode (Fig. 1A). There was no deviation from this course of infection in male BALB/c mice, demonstrating that host sex is not a critical factor in this model (Fig. 1B).

To gain a more accurate assessment of infection in defined tissues, we employed an integrated necropsy and *ex vivo* bioluminescence imaging protocol (Lewis *et al.*, 2014). Because of the size differences between tissue types, we analysed bioluminescence as fold change in radiance compared with matching tissues from uninfected controls. The data are therefore representative of parasite density in a particular tissue sample, rather than total parasite load. We found the GI tract to be the only consistently infected organ, with parasites restricted to the large intestine and/or stomach (Fig. 1C, Table S1). Approximately one third of these animals also had live parasites in the gut mesenteric tissue (Fig. 1C, Table S1). Occasional bioluminescence signals localized to other tissues besides the gut {median additional sites *n* = 1 [interquartile range (IQR) 0–2.5]}, but without any apparent tissue preference (Fig. 1C, Table S1). Imaging of the post‐necropsy carcass identified residual bioluminescence foci in some cases; notably, these were often in the skin (Fig. S2). Carcass foci were not quantified because signals could not always be unambiguously assigned to precise anatomical sites. Similar tissue‐specific infection distributions were observed in the male BALB/c–TcVI‐CLBR chronic model (Fig. 1D, Table S1). We conclude firstly that the GI tract is likely to be the only site continuously infected by TcVI‐CLBR; secondly, that active infection in diverse non‐GI sites is sporadic and transient; and thirdly, that TcVI‐CLBR infection dynamics in BALB/c mice are not influenced by host sex.

**Figure 2:**
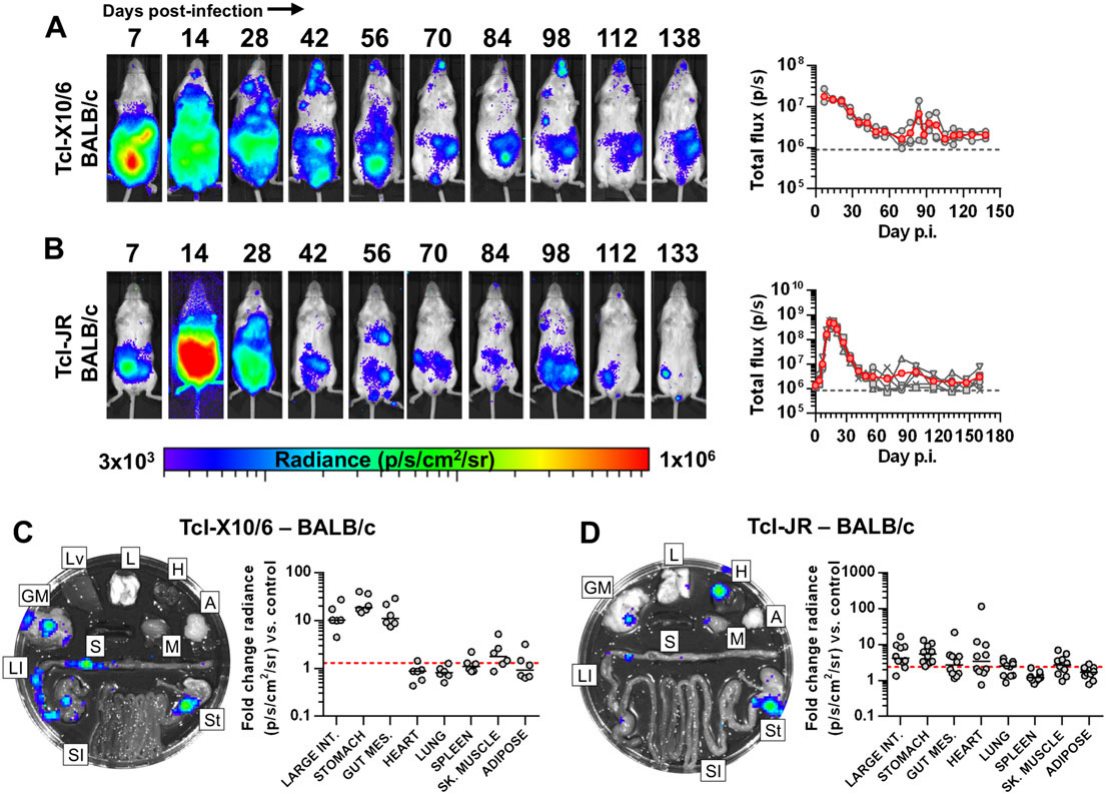
*T. cruzi* TcI infection kinetics in BALB/c mice culminate in gastrointestinal persistence and sporadic heart infection. A, B. Course of *T. cruzi* TcI‐X10/6 (*n* = 6) (A) and TcI‐JR (*n* = 10) (B) infection in BALB/c mice tracked by *in vivo* bioluminescence imaging. Panels show serial ventral images of one mouse. Log‐scale pseudocolour heat maps show intensity of bioluminescence; minimum and maximum radiances are indicated. Charts show total bioluminescence for five individual mice (grey) and their mean (red) from the experiments represented by adjacent images. Dashed line indicates detection threshold. C, D. Quantification of tissue‐specific chronic parasite densities in TcI‐X10/6 (*n* = 6, 141–146 dpi) (C) and TcI‐JR (*n* = 10, 154–161 dpi) (D) infected BALB/c mice using *ex vivo* bioluminescence imaging. Images show bioluminescence intensities for a representative mouse: Adipose (A), gut mesenteric tissue (GM), heart (H), lung (L), large intestine (LI), liver (Lv), skeletal muscle (M), spleen (S), small intestine (SI) and stomach (St). Charts show quantification of tissue‐specific infection intensities expressed as fold change in bioluminescence vs. matching tissues from uninfected controls. Black lines show median values and red dashed lines indicate detection threshold. Data are from two independent experiments.

To investigate whether chronic GI persistence is a TcVI‐CLBR‐specific phenotype or likely to be a general feature of *T. cruzi* biology, we next evaluated TcI infections in BALB/c mice. A high inoculum (2 × 10^5^ tissue culture parasites) is required to reliably obtain productive infections using the TcI‐X10/6 strain (Taylor *et al.*, 2015). This was associated with a distinctive acute bioluminescence profile lacking an expansion phase (Fig. 2A). Nevertheless, the acute dissemination pattern, and the inferred chronic parasite burden profile, as well as its focal, dynamic distribution, were found to resemble that seen for TcVI‐CLBR (*cf*. Figs 1 and 2). The tissue‐specific distribution, assessed by *ex vivo* imaging, also closely replicated the TcVI‐CLBR data – bioluminescence was observed in GI tissues in all animals chronically infected with TcI‐X10 (Fig. 2C, Table S1). Bioluminescence was detected in other sites in a subset of animals, including skeletal muscle (67%), heart (17%), spleen (17%) and adipose (33%).

Chronic BALB/c infections were also generated with TcI‐JR parasites, using 1 × 10^3^ blood trypomastigotes. The bioluminescence profile resembled a classic acute infection that resolves to a dynamic, continuously detectable chronic equilibrium, with the majority of reporter signal localized to the abdominal region (Fig. 2B). *Ex vivo* imaging again identified GI tract infections in all animals, with bioluminescence associated with 100% of stomachs, 80% of large intestines and 50% of gut mesenteries (Fig. 2D, Table S1). An average of two additional bioluminescence positive non‐GI sites were detected per animal [median additional sites *n* = 2 (IQR 1–3)], including heart (50%), skeletal muscle (60%) and lung (60%) (Fig. 2D, Table S1), as well as skin in some cases (Fig. S2). Thus, the GI tract is the primary site of persistence for all three *T. cruzi* strains tested in BALB/c mice.

### Restriction of active infection to the GI niche is mediated by the host

**Figure 3:**
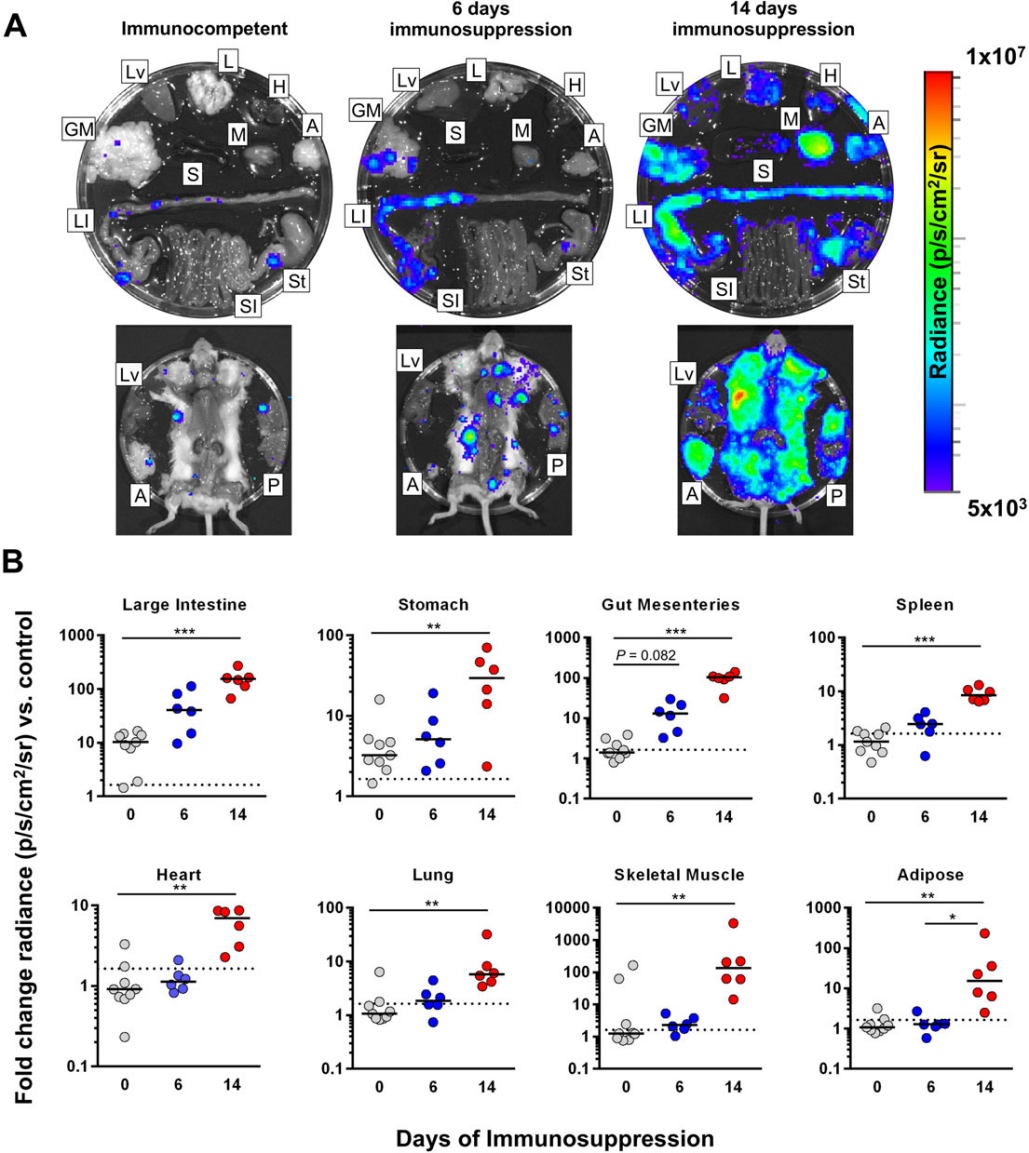
Immunosuppression leads to systemic parasite dissemination. A, B. Tissue‐specific chronic TcVI‐CLBR parasite distributions and densities in BALB/c mice after 6 (*n* = 6) or 14 (*n* = 6) days of cyclophosphamide‐induced immunosuppression compared with immunocompetent controls (*n* = 9). Representative images (A) show *ex vivo* bioluminescence intensities for samples from a representative mouse in each group: Adipose (A), gut mesenteric tissue (GM), heart (H), lung (L), large intestine (LI), liver (Lv), skeletal muscle (M), peritoneum (P), spleen (S), small intestine (SI) and stomach (St). Log‐scale heat maps indicate bioluminescence intensity; minimum and maximum radiances for pseudocolour scale are indicated. Charts (B) show infection intensities expressed as fold change in bioluminescence intensity compared with matching tissues from uninfected controls. Black lines indicate median values. Dashed lines indicate background luminescence cut‐offs equal to the mean + 2 SD of the fold change in bioluminescence intensity for empty regions of interest (ROI) compared with empty ROI in images obtained for uninfected mice. Asterisks indicate *p*‐values for Kruskal–Wallis tests between groups (*P < 0.05; **P < 0.01; ***P < 0.001). Data are from two independent experiments.

Long‐term restriction, whether relative or absolute, of *T. cruzi* to a gut niche could be a consequence of parasite tropism or differential immune responses between sites. The TcVI‐CLBR strain infects virtually all tissues during the acute phase in BALB/c mice (Lewis *et al.*, 2014), favouring the host response as the critical mediator. Tissue tropism may not be a fixed property of *T. cruzi* in different phases though, and previous data do not provide evidence directly relevant to the chronic phase. To address whether host immunity is required for the tissue‐specific distribution of chronic infection, we used cyclophosphamide to immunosuppress BALB/c mice with established TcVI‐CLBR infections. This treatment has cytotoxic effects on both B and T lymphocytes and leads to a rapid increase in total bioluminescence (Francisco *et al.*, 2015). *Ex vivo* imaging revealed that after six days immunosuppression, there was increased bioluminescence intensity in the large intestine and gut mesenteries (Fig. 3). By day 14, parasite loads were significantly increased in all tissues sampled (Fig. 3). These data indicate that the relative restriction of active *T. cruzi* infection to the gut in chronically infected mice is explained by tissue‐specific host responses, rather than tissue tropism of the parasite.

### Broadly conserved GI parasite persistence and model‐dependent systemic distributions

**Figure 4:**
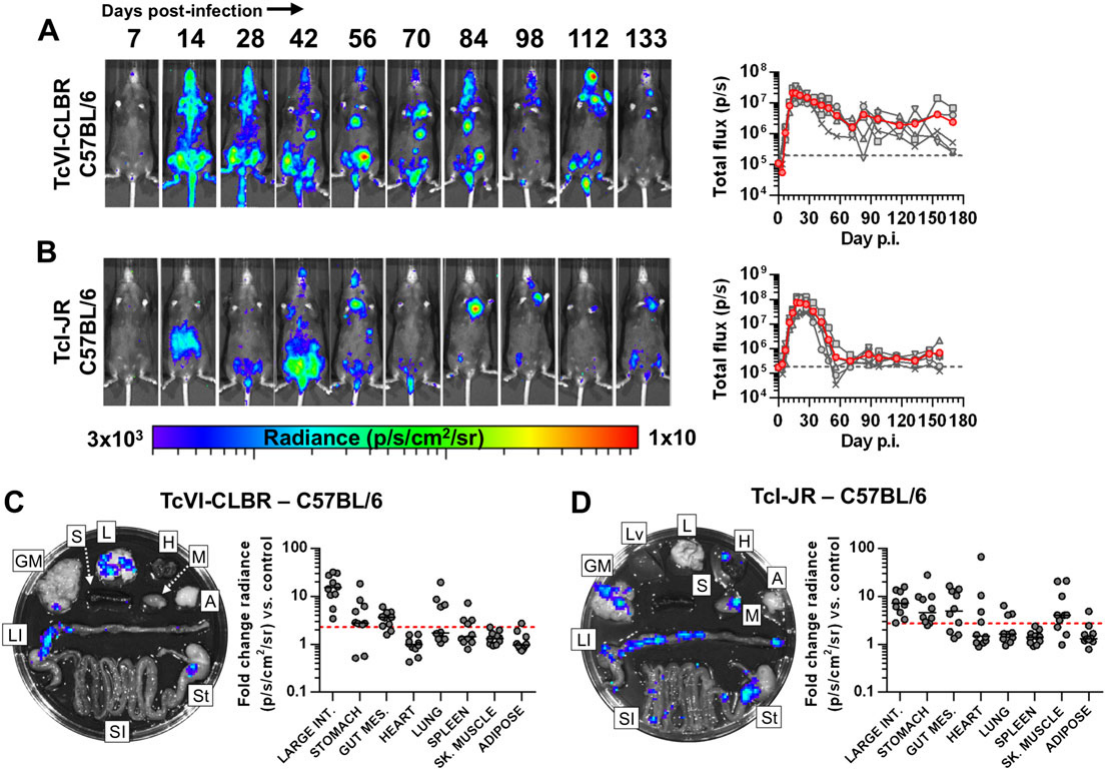
*T. cruzi* TcI‐JR and TcVI‐CLBR have similar infection dynamics in C57BL/6 mice including chronic gut persistence. A, B. Course of *T. cruzi* TcVI‐CLBR (*n* = 10) (A) and TcI‐JR (*n* = 10) (B) infection in C57BL/6 mice tracked by *in vivo* bioluminescence imaging. Panels show serial ventral images of one mouse. Log‐scale pseudocolour heat‐maps show intensity of bioluminescence; minimum and maximum radiances are indicated. Charts show total bioluminescence for five individual mice (grey) and their mean (red) from the experiments represented by adjacent images. Dashed line indicates detection threshold. C, D. Quantification of tissue‐specific chronic parasite densities in TcVI‐CLBR (*n* = 10, 170–175 dpi) (C) and TcI‐JR (*n* = 10, 156–158 dpi) (D) infected C57BL/6 mice using *ex vivo* bioluminescence imaging. Images show bioluminescence intensities for a representative mouse: Adipose (A), gut mesenteric tissue (GM), heart (H), lung (L), large intestine (LI), liver (Lv), skeletal muscle (M), spleen (S), small intestine (SI) and stomach (St). Charts show quantification of tissue‐specific infection intensities expressed as fold change in bioluminescence versus matching tissues from uninfected controls. Black lines show median values and red dashed lines indicate detection threshold. Data are from two independent experiments.

To investigate the influence of host genetic diversity on *T. cruzi* infection dynamics, we compared TcI‐JR and TcVI‐CLBR parasites in BALB/c, C57BL/6 (B6) and C3H/HeN (C3H) mice. All B6 mice developed chronic infections with both parasite strains that could be tracked by *in vivo* imaging (Fig. 4A and B). The bioluminescence intensity profile for TcVI‐CLBR infections indicated a more gradual acute‐chronic transition than for TcI‐JR. Otherwise, there were no major differences in the spatiotemporal distributions of bioluminescence foci between strains. We refrained from making quantitative comparisons between mouse types, because coat colour influences bioluminescence detection. Post‐mortem organ imaging showed that parasite distributions in B6 were remarkably similar to those seen in BALB/c hosts: The large intestines of all B6 mice infected with either parasite strain were bioluminescence positive, and most had concomitant signals in stomach (TcI‐JR 90%, TcVI‐CLBR 80%) and/or gut mesentery (TcI 70%, TcVI 80%) (Fig. 4C and D, Table S1). As in BALB/c mice, the frequency of non‐GI infected sites was higher for TcI‐JR than TcVI‐CLBR: median additional sites *n* = 1 [IQR 0.75–2.25] versus 0.5 [IQR 0–2] respectively. Of those sites, TcI‐JR was most often detected in skeletal muscle (70%) and heart (40%), whereas TcVI‐CLBR was never detected in these sites and instead favoured lung (40%) and spleen (40%). Some residual carcasses from both TcI‐JR and TcVI‐CLBR infected B6 contained minor additional bioluminescence foci, often localized to the skin, but these were not quantified (Fig. S2).

**Figure 5:**
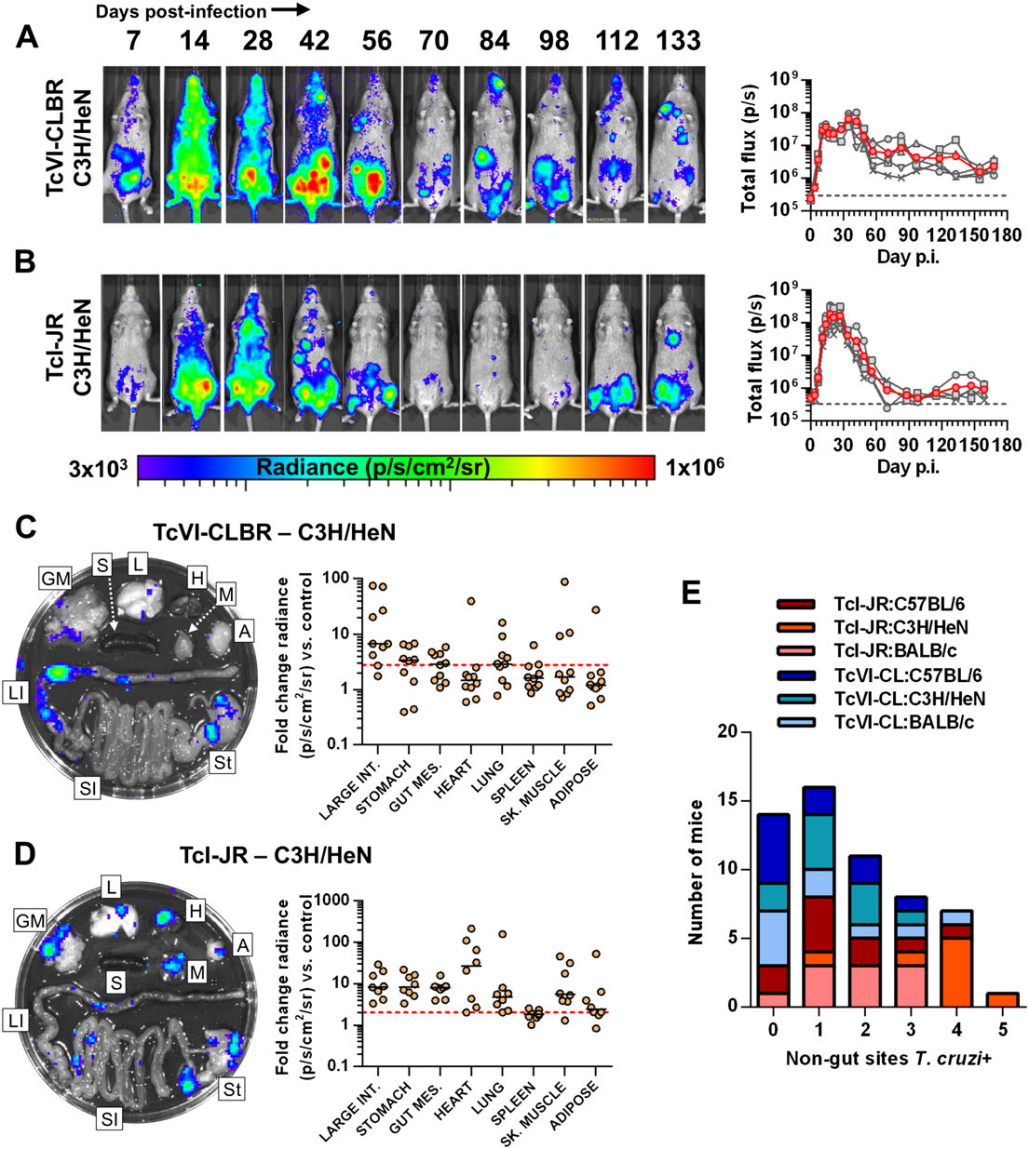
Distinct *T. cruzi* TcI‐JR and TcVI‐CLBR infection dynamics in C3H/HeN mice. A, B. Course of *T. cruzi* TcVI‐CLBR (*n* = 10) (A) and TcI‐JR (*n* = 10) (B) infection in C3H/HeN mice tracked by *in vivo* bioluminescence imaging. Panels show serial ventral images of one mouse. Log‐scale pseudocolour heat maps show intensity of bioluminescence; minimum and maximum radiances are indicated. Charts show total bioluminescence for five individual mice (grey) and their mean (red) from the experiments represented by adjacent images. Dashed line indicates detection threshold. C, D. Quantification of tissue‐specific chronic parasite densities in TcVI‐CLBR (*n* = 10, 154–160 dpi) (C) and TcI‐JR (*n* = 8, 168–174 dpi) (D) infected C3H/HeN mice using *ex vivo* bioluminescence imaging. Images show bioluminescence intensities for a representative mouse: Adipose (A), gut mesenteric tissue (GM), heart (H), lung (L), large intestine (LI), liver (Lv), skeletal muscle (M), spleen (S), small intestine (SI) and stomach (St). Charts show quantification of tissue‐specific infection intensities expressed as fold change in bioluminescence vs. matching tissues from uninfected controls. Black lines show median values and red dashed lines indicate detection threshold. E: Number of infected tissues outside the GI tract compared with equivalently infected BALB/c, and C57BL/6 mice at experimental end‐points. Hearts, lungs, spleens, adipose and skeletal muscle samples above threshold *ex vivo* bioluminescence were scored as *T. cruzi* positive. Data are from two independent experiments.

C3H mice inoculated with TcI‐JR or TcVI‐CLBR had an extended acute phase, with bioluminescence reaching chronic equilibrium 2–3 weeks later than equivalently infected BALB/c (Fig. 5A and B). Much of the acute parasite burdens localized to the abdomen. Predominance of spatiotemporally dynamic abdominal foci was observed for both strains in the chronic phase (Fig. 5A and B). These findings were not confounded by the i.p. route of inoculation because dorsal subcutaneous inoculations generated similar results (Fig. S3), as was previously shown for TcVI‐CLBR in BALB/c (Lewis *et al.*, 2014). Additional foci were frequently observed at diverse (non‐abdominal) anatomical sites, but without any apparent parasite strain‐specific quantitative or spatial trend (Fig. 5). Post‐mortem imaging again identified the GI tract as the predominant site of parasite persistence for both TcI‐JR and TcVI‐CLBR (100% and 90% large intestine or stomach positive respectively) (Fig. 5C and D, Table S1). TcI‐JR was present in non‐GI samples at a significantly higher frequency than TcVI‐CLBR: median additional sites *n* = 4 [IQR 3.25–4] versus *n* = 1 [0.75–2] respectively, Mann–Whitney test, *U* = 6.5, *p* = 0.0013 (Fig. 5E). Heart, lung and skeletal muscle were bioluminescence positive in a large subset of animals, and the inferred relative infection intensities were in the same range as that seen for the gut (Fig. 5, Table S1). Conversely, for TcVI‐CLBR, the differential between GI and non‐GI tissue infection frequencies was similar to BALB/c and B6 – parasites were detected sporadically in diverse sites ranging from lung (60% of mice) to skeletal muscle (30%) to heart and adipose (both 10%) (Fig. 5, Table S1).

In summary, we have identified key similarities and differences in *T. cruzi* infection dynamics for different host‐parasite strain combinations. The GI tract, primarily the large intestine and secondarily the stomach, was the only consistent site of active chronic *T. cruzi* infection in all models tested. Restriction to the gut was not absolute in any strain pairing, as shown by the presence of bioluminescence foci in diverse additional sites. There was a trend towards a higher frequency of infection in ‘systemic’ sites outside the gut for TcI‐JR compared with TcVI‐CLBR, significantly so in C3H mice. Heart‐specific infection was detected in ≤50% of mice in all cases, with the exception of TcI‐JR–C3H, in which the rate was 88%. However, lung and skeletal muscle had similar and often higher infection rates compared with the heart. We conclude that while none of the strains tested has any intrinsic tissue tropism in established infections, host‐parasite genotype combinations do shape frequencies of transient infection in sites outside the gut, including the heart.

### Model‐dependent cardiac disease pathology

**Figure 6:**
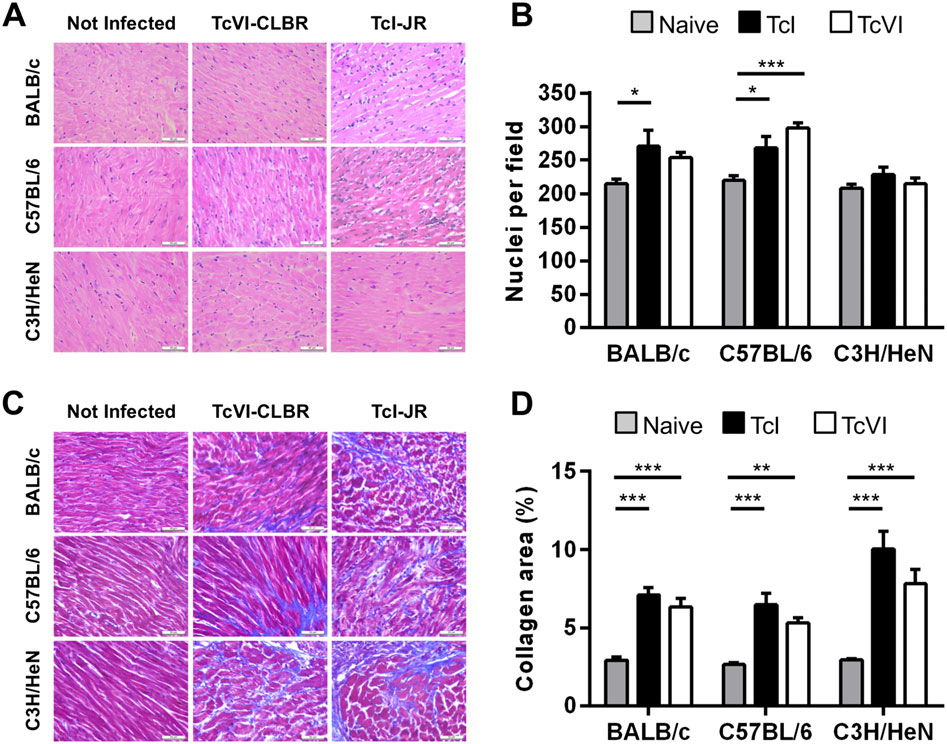
Variable chronic cardiac pathology across host‐parasite strain combinations. A. Representative myocardial sections stained with haematoxylin‐eosin, magnification 400×, scale bar = 50 µm. B. Quantitative histopathological analysis of myocardium samples obtained at 154–174 days of *T. cruzi* infection from the following groups: TcVI‐CLBR‐BALB/c (*n* = 9), TcVI‐CLBR‐C57BL/6 (*n* = 10), TcVI‐CLBR‐C3H/HeN (*n* = 10), TcI‐JR‐BALB/c (*n* = 10), TcI‐JR‐C57BL/6 (*n* = 10), TcI‐JR‐C3H/HeN (*n* = 8), uninfected control BALB/c, C57BL.6 and C3H/HeN (all *n* = 10). Number of nuclei per 6 × 10^4^ µm^2^ were quantified as a marker of the extent of cellular infiltration. C. Representative myocardial sections stained with Masson's trichrome, magnification 400×, scale bar = 50 µm. D. Quantification of collagen content (% blue area in Masson's trichrome stained sections) as a marker of cardiac fibrosis severity in same groups as in (B). Data are the means + SEM and are from two experiments. Asterisks indicate *p*‐values for one way ANOVA comparisons between groups (*P < 0.05; **P < 0.01; ***P < 0.001).

To assess chronic heart disease development, we evaluated myocardial tissue inflammation and fibrosis as markers of pathology. Quantitative histopathological analysis revealed mild but significant diffuse cellular infiltration in the TcI‐JR–BALB/c, TcI‐JR–C57BL/6 and TcVI‐CLBR–C57BL/6 infection models (Fig. 6A and B). Inflammatory infiltrates comprised predominantly mononuclear cells with lymphocytic morphology. No parasites were seen in any of the tissue sections used for analysis. Nevertheless, there were highly significant increases in the collagen content – indicative of pathological fibrosis – in all six host–parasite genotype combinations (Fig. 6C and D). When present, fibrosis was diffusely distributed within the myocardium in all cases. In C3H mice infected with either strain, fibrosis was also present as focal deposits and was more extensively distributed between the muscle fibres than in BALB/c and B6 models. No differences in inflammation or fibrosis between atrial and ventricular regions were apparent.

**Figure 7:**
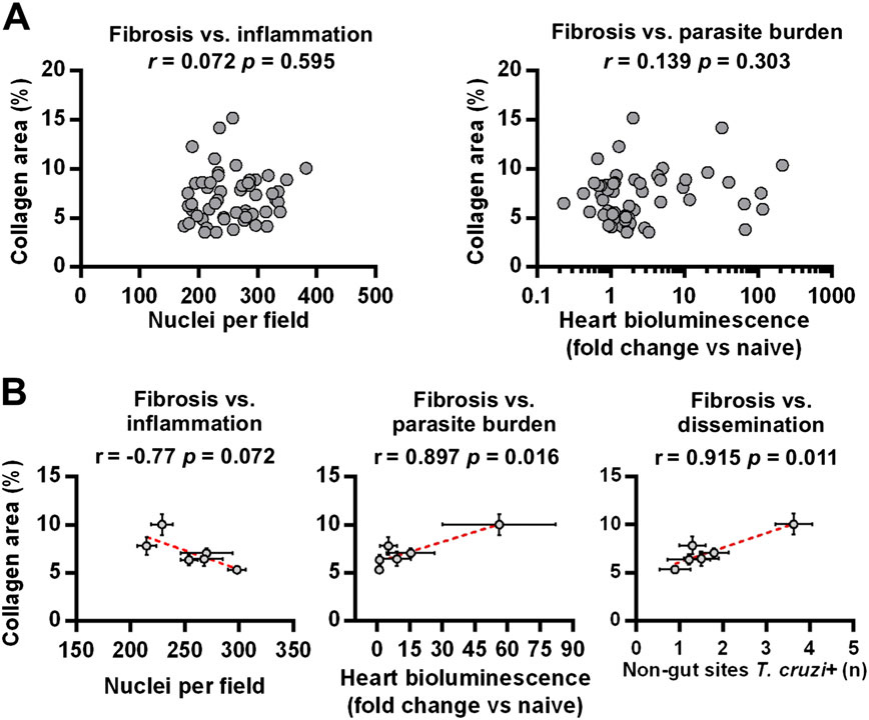
Correlates of chronic heart pathology in *T. cruzi* infected mice. A. Pearson correlation analysis of myocarditis scores (left) and cardiac end‐point parasite densities (*ex vivo* bioluminescence intensities) (right) against cardiac fibrosis scores. Data are from samples obtained at 154–174 days of *T. cruzi* infection from the following groups: TcVI‐CLBR‐BALB/c (*n* = 9), TcVI‐CLBR‐C57BL/6 (*n* = 10), TcVI‐CLBR‐C3H/HeN (*n* = 10), TcI‐JR‐BALB/c (*n* = 10), TcI‐JR‐C57BL/6 (*n* = 10) and TcI‐JR‐C3H/HeN (*n* = 8). B. Pearson correlation analysis of group mean cardiac fibrosis scores against group mean myocarditis scores (left), cardiac end‐point parasite densities (*ex vivo* bioluminescence intensities) (middle) and number of *ex vivo T. cruzi* bioluminescence positive non‐gut tissues as an indicator of dissemination level (right). Data are from the same groups as in (A). Error bars show SEM. Dashed lines are linear regression models.

There was no support for an association between cardiac fibrosis severity and either end‐point inflammation or local tissue infection intensities in individual animals (Fig. 7A). However, at the group average level, which should better represent the dynamics of ongoing infections, there was a trend towards a negative association with myocarditis and a significant positive association with heart‐specific parasite loads (Fig. 7B). The latter finding was dependent on the TcI‐JR‐C3H data, which exhibited the most severe fibrosis and the highest average heart bioluminescence intensities. Together, these data indicate that heart‐specific damage accumulates according to the frequency of transiently active parasitism in this organ, rather than developing as a result of continuous local infection. Consistent with this, we also identified a significant correlation between heart fibrosis severity and the overall number of actively infected organs outside the gut, used as a measure of dissemination (Fig. 7B).

To compare the relative influence of the parasite strain and host background on heart pathology, a two‐way ANOVA analysis of the cardiac fibrosis scores for each model was performed. Both parasite genotype (*p* = 0.024) and mouse genotype (*p* = 0.0004) were significant sources of variation in the data, but the latter parameter explained a greater proportion of fibrosis variability: 7.3% versus 25.0% respectively. There was no support for interaction between the two factors (1.5%, *p* = 0.57).

Several conclusions can be drawn from these data. *Trypanosoma cruzi* caused chronic cardiomyopathy resembling human Chagas disease, irrespective of parasite or host genetic background. The severity of cardiac fibrosis was dependent on the host–parasite strain combination and was significantly greater in pairings associated with a higher mean local infection intensity, as well as low levels of inflammation. A lesser capacity to restrict active infection to the gut in a host–parasite genotype combination had predictive value for heart disease severity.

## Discussion

The aim of this study was to evaluate the interaction between *T. cruzi* infection dynamics and the pathogenesis of experimental chagasic cardiomyopathy. To address this, we applied sensitive, minimally biased, real‐time imaging tools and quantitative histopathological analysis. Our approach was designed to assess host and pathogen genetic diversity, which are extensive in nature, and to test whether these factors affect the course and outcome of infection. The limit of detection of our bioluminescence imaging system is close to 100 parasites when they are freely dispersed in the peritoneal cavity (Lewis *et al.*, 2014). Thus, the interpretation that we offer cannot completely exclude the possibility that extremely low numbers of parasites remain in bioluminescence negative samples. Furthermore, quiescent *T. cruzi* (akin to *Plasmodium* hypnozoites), though never described, would not generate bioluminescence if they were present, so we restrict ourselves to discussion of active as opposed to latent infection. Previously, we observed an excellent correlation between *ex vivo* bioluminescence intensity and quantitative PCR (qPCR) targeting *T. cruzi* DNA (Lewis *et al.*, 2014), which was consistent between tissue types, indicating that signal intensity is not site‐dependent. However, we have also shown that despite its high sensitivity (approximately one parasite per 10^6^ host cells), the reliability of qPCR as a detection method in chronic infections is limited by the highly focal nature of parasite distribution (Francisco *et al.*, 2015).

### The gut is the primary reservoir of chronic *T. cruzi* infection in mice

We previously found that TcVI‐CLBR infections are initially widely disseminated in BALB/c mice, but become confined almost exclusively to GI tissue during the chronic phase (Lewis *et al.*, 2014). In this current study, we conducted long‐term infection tracking experiments for TcVI‐CLBR and two strains from the diverse TcI lineage. The range of TcI extends from the United States to Argentina, and it is the predominant cause of human infection in Ecuador, Colombia, Venezuela and North America, where digestive pathologies are absent (Miles *et al.*, 1981). Nevertheless, in BALB/c, B6 and C3H mice, the large intestine and stomach were the primary reservoir sites of active infection for both TcI strains and for TcVI‐CLBR. Thus, GI tissues are a permissive niche for active *T. cruzi* infection, irrespective of the host or parasite genetic diversity represented in our models. The TcI and TcVI strains are highly divergent, ~7% at the mitochondrial nucleotide sequence level (Lewis *et al.*, 2011), so features conserved between them, in this case GI persistence, are probably intrinsic to *T. cruzi* biology.

Data on the presence of *T. cruzi* in the human gut is limited. Histological studies identified persistence in GI tissue in 20–50% of megaesophagus cases (Adad *et al.*, 1991; de Castro Côbo *et al.*, 2012), but using PCR others found parasite DNA in 100% of such samples (da Silveira *et al.*, 2005). Nevertheless, anti‐parasitic chemotherapy is not considered justifiable for seropositive individuals with digestive symptoms but normal heart function (Bern, 2011). This is largely because of lack of evidence for treatment efficacy against digestive disease, but is also influenced by the prevailing view that megasyndromes are the result of denervation during the acute phase (Köberle, 1970; de Oliveira *et al.*, 1998). Counter to this, our finding of long‐term parasite persistence suggests that local infection could potentially influence the development of digestive disease into the chronic phase.

The relative restriction of *T. cruzi* to the gut is mediated by ongoing host responses, as shown by dramatic expansion of disseminated parasite loads under immunosuppressive conditions. This indicates important differences between systemic and GI immune responses, which could variously affect parasite survival, replication or migratory potential. The hypo‐inflammatory microenvironment necessary for preservation of gut barrier integrity (Macpherson and Smith, 2006; Murai *et al.*, 2009) may help form a permissive niche for parasite persistence. The early increase in GI tissue infection intensity following initiation of immunosuppression shows that the host actively suppresses *T. cruzi* below a certain threshold. Yet there is clearly regional variation in permissiveness, because *T. cruzi* was always found in the large intestine and/or stomach, but only occasionally in the small intestine. This could be a result of regional differences in lymphocyte phenotypes (Mowat and Agace, 2014), or in the availability of permissive host cell subsets, such as specialized enteric myocytes or mononuclear phagocytes.

### Transient heart infection and pathology

Containment to the gut was not absolute in any strain pairing, and this is consistent with the requirement to maintain vector transmission potential. We often observed parasite foci in the skin and other non‐GI tissues, but importantly the frequency of active infection in any one tissue type did not approach the consistency seen for the large intestine or stomach. The scarcity and apparent non‐persistence of these systemic foci suggests that mice of all types tested can mount effective anti‐parasite responses outside the gut. Indeed, *T. cruzi*‐specific CD8^+^ T cells, with unimpaired cytotoxic function, have been described in B6 mice with long‐term established infections (Bustamante *et al.*, 2008). Nevertheless, their effectiveness may still decline in decades‐long human infections (Albareda *et al.*, 2013). The use of xenodiagnosis and transmission by organ transplantation or blood transfusion (Schmunis and Cruz, 2005) shows that parasites can, at least temporarily, access the blood and peripheral tissues in chronic human infections. We propose that a second equilibrium occurs in the periphery, including the heart, which only permits discrete and transient episodes of replicative infection. Our data suggest that host‐parasite genotype combinations influence the frequency of these episodes and hence local pathogenesis.

Chronic *T. cruzi* infection was associated with fibrotic heart disease in all our models. A high degree of variation in fibrosis severity was observed between individual mice, but this did not correlate with end‐point cardiac parasite loads or myocarditis intensity. This is understandable if active heart parasitism is episodic, rather than continuous. Indeed, when we examined group‐level correlates, which should better reflect intermittent processes compared with end‐point measures, there was a clear association between fibrosis severity and both the relative capacity to restrict active infection to the GI tissues and the frequency of heart‐specific infection.

We found that parasite genotype had a small but significant impact on disease severity: in all hosts TcI‐JR infection resulted in more severe cardiac fibrosis than infection with TcVI‐CLBR. The extent of intra‐lineage genetic diversity, particularly within TcI, means that these differences should not be extrapolated to suggest that TcI is, in general, more virulent than TcVI. The potential for strain‐dependent variation in chronic cardiomyopathy presentation and severity is known to exist, even between closely related parasites (Schlemper *et al.*, 1983; Andrade, 1990). In this study, the infection dynamics of TcI‐JR and TcI‐X10 varied in some respects and other TcI‐X10 clones are known to differ in virulence (Postan *et al.*, 1983). We detected live TcVI‐CLBR in heart tissue in only ~10% of chronically infected BALB/c. Quantitative PCR data from other studies support the presence of DNA of other TcVI strains in heart samples in similar experiments, at least at the group mean level (Cencig *et al.*, 2011; Rodriguez *et al.*, 2014). Some heterogeneity may therefore also exist within the hybrid TcVI lineage, despite its very low genetic diversity (Lewis *et al.*, 2011).

In this study, host genotype was found to have a stronger influence on pathogenesis than that of the parasite. The finding that heart infection frequencies are coupled to pathogenesis was largely dependent on the major difference between C3H mice infected with TcI‐JR and other strain pairings. These mice exhibited the most disseminated active infection foci, highest mortality rate and highest cardiac fibrosis scores, whereas, for example, TcVI‐infected B6 displayed comparatively efficient gut restriction (or systemic control), 100% survival and more moderate fibrosis. The C3H background has previously been associated with relatively severe pathology, which resembles several features of human Chagas disease (Postan *et al.*, 1987; Marinho *et al.*, 2009; Daliry *et al.*, 2014) This has not proven fully reproducible between laboratories (Eickhoff *et al.*, 2010), perhaps because of the use of different C3H sub‐strains. Comparative studies with mice considered relatively resistant (e.g. B6) have implicated several factors in survival of acute infection, such as IgG isotype profile (Rowland *et al.*, 1992) and production of IL‐10 (Roffê *et al.*, 2012). However, the significance of such findings for chronic pathogenesis is yet to be demonstrated. In any case, defining cause and effect relationships between parasite loads, immunological markers and disease severity will be an ongoing challenge. A contributory factor has been the lack of sensitive, unbiased methods to determine tissue parasite loads. As shown here, application of bioluminescence imaging approaches to quantify living parasites in tissues should now substantially improve our ability to investigate the mechanism of chronic pathogenesis.

It is important to note that analysis of myocarditis and cardiac fibrosis in chronically infected mice does not comprehensively model human disease. For example, cardiac arrhythmias are typical in cardiac cases, and blood clot formation accounts for 10–15% of all Chagas disease deaths (Rassi *et al.*, 2001). Digestive megasyndromes are not reproduced in mice. It was not an aim of this study to address these aspects of pathology, and we focussed on inflammation and fibrosis, which are readily quantifiable. Although these phenomena are considered to be proximal drivers of arrthymias and microvascular alterations (Marin‐Neto *et al.*, 2007; Healy *et al.*, 2015), further studies will be necessary to establish a direct contribution of genetics and infection dynamics to these other aspects of Chagas disease.

### Coupling parasite dynamics to cardiac pathogenesis

The framework that we outline for a link between the frequency of active heart infection episodes and pathogenesis suggests two non‐exclusive underlying scenarios for parasite dynamics. In the chronic phase, there may be parasites that are very few in number or in a quiescent form, continuously present in cardiac tissue, that sporadically undergo expansion or activation to generate detectable bioluminescence. Our original observations of TcVI‐CLBR in BALB/c, in which heart parasites are rarely detected but disease is still evident, led us to propose an alternative model whereby sporadic trafficking of parasites into the heart could explain the pathology in the absence of local persistence (Lewis *et al.*, 2014). There are several reasons to conclude that this latter scenario is on balance the more likely. Firstly, highly sensitive qPCR fails to detect *T. cruzi* DNA in bioluminescence negative cardiac tissue such that latent parasite loads would need to be below the qPCR limit of detection of ~1 parasite per 10 mg (Francisco *et al.*, 2015). Secondly, we could find no histological evidence in multiple sections of cardiac tissue in any of these mice and reports of such observations by others are extremely rare. Thirdly, peripheral infection foci in the TcVI‐CLBR–BALB/c model typically appear and disappear over the course of a single day, consistent with trafficking of infected host cells (Lewis *et al.*, 2014). Fourthly, in a fatal acute infection model, parasite burdens in the heart depended on the ability of *T. cruzi* to replicate specifically in myeloid cells expressing *Slamf1* (Calderón *et al.*, 2012), which has a pro‐migratory function (van Driel *et al.*, 2012; Wang *et al.*, 2015). Our new observations on the timing of immunosuppression‐induced dissemination indicate that systemic tissue infections may be seeded from the parasite reservoir in the GI tissues. However, our approach did not allow direct tracking of individual foci, so we cannot completely exclude the possibility that cyclophosphamide treatment led to local reactivation of quiescent parasites in systemic sites that required >6 days to exceed the detection threshold. Further studies will be required to establish whether *T. cruzi* latency within or trafficking into the heart is the dominant mode of infection driving local pathogenesis. Nevertheless, the insight that cardiac pathology could be caused by repeatedly transient infection, rather than by continuous local persistence, has the potential to reconcile many of the controversies surrounding the role of *T. cruzi* in cardiac pathogenesis. It may also help to explain the clinical heterogeneity associated with human infections.

Interestingly, the heart was not preferentially infected compared with other tissues, such as lung and skeletal muscle. Explanations for the heart being particularly susceptible to progressive, life‐threatening disease include the low regenerative capacity of adult myocardium (Porrello *et al.*, 2011), molecular mimicry between *T. cruzi* and heart autoantigens (Cunha‐Neto *et al.*, 1996) and the unique importance of neurological homeostasis in the heart. Irrespective of the downstream pathogenic mechanism(s), our data indicate that *T. cruzi* is the ultimate driver of chronic myocardial fibrosis, but one that does not have to be continually present in the heart. This reinforces the validity of pursuing new anti‐parasitic drugs and should contribute to a better informed assessment of their efficacy and PKPD requirements. Finally, the possibility that the gut is an immunologically privileged site for *T. cruzi* in humans has important implications for Chagas disease vaccine and immunotherapy development.

## Experimental procedures

### Ethics statement

All animal work was carried out under UK Home Office project licence (PPLs 70/6997 and 70/8207) and was approved by the London School of Hygiene and Tropical Medicine Animal Welfare and Ethical Review Board. All protocols and procedures were conducted in accordance with the UK Animals (Scientific Procedures) Act 1986 (ASPA).

### Parasites

*Trypanosoma cruzi* strains CL Brener (CLBR), Silvio X10/6 (X10), JR cl4 (JR), were grown as epimastigotes at 28°C in RPMI‐1640 medium supplemented with 0.5% (w/v) tryptone, 20 mM HEPES pH 7.2, 30 mM haemin, 10% heat‐inactivated fetal bovine serum, 2 mM sodium glutamate, 2 mM sodium pyruvate, 100 µg ml^−1^ streptomycin and 100 U ml^−1^ penicillin. Metacyclic trypomastigotes (MTs) were obtained by transfer to Graces‐IH transformation medium (Isola *et al.*, 1986). MTs were harvested after 4–7 days, when typically 70–90% of parasites had differentiated. Tissue culture trypomastigotes were obtained from infected L6 rat skeletal myoblasts grown at 37°C and 5% CO_2_ in RPMI‐1640 medium supplemented with 20 mM HEPES pH 7.2, 10% heat‐inactivated fetal bovine serum, 2 mM sodium glutamate, 2 mM sodium pyruvate, 100 µg ml^−1^ streptomycin and 100 U ml^−1^ penicillin. Bioluminescent CLBR and X10 parasites constitutively expressing the firefly luciferase *PpyRE9h* (Branchini *et al.*, 2010) were described previously (Lewis *et al.*, 2014; Taylor *et al.*, 2015). The TcI‐JR cell line expressing *PpyRE9h* was generated by genomic integration into the rRNA locus (Lewis *et al.*, 2014). Genotypes of wild type and bioluminescent lines were confirmed at three independent loci (Lewis *et al.*, 2009).

### *In vitro* assays

The Luciferase Assay System (Promega) was used to screen transfected *T. cruzi* clones for luciferase expression (Lewis *et al.*, 2014). Parasite lysates were mixed with the assay substrate, and luminescence was measured immediately using a SpectraMax® M3 microplate reader (Molecular Devices). To assess luciferase expression in individual amastigotes, L6 myoblasts grown on glass coverslips were processed for immunofluorescence analysis 5 days after infection. Coverslips were fixed in 2% paraformaldehyde in PBS, permeabilized using 0.1% Triton X‐100, then stained with 1:500 goat anti‐luciferase pAb (Promega) followed by 1:1000 Alexa488 conjugated donkey anti‐goat IgG (Invitrogen). DNA was labelled using 1 μM Hoechst 33342, before mounting with FluorPreserve (Calbiochem). Images were acquired on an LSM 510 confocal microscope (Zeiss).

### Mice and infections

BALB/c, C57BL/6 and C3H/HeN mice were purchased from Charles River (UK), and CB17 SCID mice were bred in‐house. Animals were maintained under specific pathogen‐free conditions in individually ventilated cages. They experienced a 12 h light/dark cycle and had access to food and water *ad libitum*. Female mice aged 8–12 weeks were used, except where otherwise stated. SCID mice were infected with 1 × 10^4^ *in vitro*‐derived tissue culture trypomastigotes in 0.2 ml PBS via i.p. injection. Unless otherwise stated, all other mice were infected by i.p injection of 1 × 10^3^ BTs derived from parasitaemic SCID mouse blood. All infected SCID mice developed fulminant infections and were euthanized at or before humane end‐points. At experimental end‐points, mice were sacrificed by ex‐sanguination under terminal anaesthesia. In some experiments chronically infected mice (>150 days post infection) were immunosuppressed with cyclophosphamide (200 mg kg^−1^ day^−1^) by i.p. injection at 3–4 day intervals, for a maximum of three doses (Francisco *et al.*, 2015).

### Bioluminescence imaging

Mice were injected with 150 mg kg^−1^ d‐luciferin i.p., then anaesthetized using 2.5% (v/v) gaseous isoflurane in oxygen. To measure bioluminescence, mice were placed in an IVIS Lumina II system (Caliper Life Science) and images were acquired 10–20 min after d‐luciferin administration using LivingImage 4.3. Exposure times varied between 30 s and 5 min, depending on signal intensity. After imaging, mice were revived and returned to cages. For *ex vivo* imaging, mice were injected with 150 mg kg^−1^ d‐luciferin i.p., then sacrificed by ex‐sanguination under terminal anaesthesia 7 min later. Mice were perfused with 10 ml 0.3 mg ml^−1^ d‐luciferin in PBS via the heart. Organs and tissues were imaged in two stages. Firstly, heart, lungs, spleen, liver, gastrointestinal tract, gastrointestinal mesenteric tissue, a skeletal muscle sample (quadriceps) and a visceral fat sample were transferred to a Petri dish in a standardized arrangement, soaked in 0.3 mg ml^−1^ d‐luciferin in PBS and then imaged using maximum detection settings (5 min exposure, large binning). Then the remaining animal parts were checked for residual bioluminescence foci in the second stage of *ex vivo* imaging, also using maximum detection settings.

To estimate parasite burden in live mice, regions of interest (ROIs) were drawn using LivingImage v.4.3 to quantify bioluminescence expressed as total flux (photons/second) summed from dorsal and ventral images. The detection threshold for *in vivo* imaging was determined using control uninfected mice. To quantify infection intensities in *ex vivo* tissues, individual ROIs were drawn to quantify bioluminescence expressed as radiance (photons s^−1^ cm^−2^ sr^−1^). Because different tissue types from uninfected control mice have different background radiances, we normalized the data from infected mice using matching tissues from uninfected controls and used the fold‐change in radiance, compared with these tissue‐specific controls, as the final measure of *ex vivo* bioluminescence. The detection threshold for *ex vivo* imaging was estimated using the fold‐change in radiance for empty ROIs in images obtained for infected mice compared with matching empty ROIs in images for uninfected control mice.

### Histopathology

Tissue samples were fixed in GlyoFixx (Thermo Scientific) for 24–72 h, dehydrated, cleared and embedded in paraffin. Three micron heart sections were stained with hematoxylin and eosin (H&E) or Masson's trichrome and 15 400× magnification images of randomly selected fields covering the ventricular and atrial regions were taken on a Leica DM3000 microscope (40× objective) for quantitative histomorphometric analysis. The base of the heart and major vessels were excluded because of high inherent collagen content. The number of images required for accurate quantification representative of the whole section of myocardium was determined by a stability analysis of measurements based on a range of fields from a minimum of 5 to a maxiumum of 20. An index of cellular infiltration was derived by quantifying the number of nuclei in images of H&E stained sections. An increase in the number of nuclei compared with uninfected controls was considered indicative of myocarditis. A fibrosis index was derived by quantifying the area of collagen (blue pixels) in images of trichrome stained sections. An increase in collagen content compared with uninfected controls was considered indicative of cardiac fibrosis. All images were analysed using Leica Application Suite v4.5.0.

### Statistics

Individual animals were used as the unit of analysis, except where otherwise stated. No blinding or randomization protocols were used. Statistical differences between groups were evaluated using the Mann–Whitney *U*‐test, one‐way or two‐way ANOVA with Bonferroni post‐hoc correction or the Kruskal–Wallis test with Dunn's post‐hoc correction. Pearson correlation analysis was used to evaluate relationships between variables. These tests were performed in GraphPad Prism v.6. Differences of *p* < 0.05 were considered significant.

## Supporting information


**Table S1**. Summary of tissue‐specific chronic infection frequencies across *T. cruzi* – mouse strain combinations.
**Fig. S1**. Luciferase expression in *T. cruzi* strain TcI‐JR.
**Fig. S2**. Residual foci of *T. cruzi* infection frequently localized to skin.
**Fig. S3**. Infection dynamics for *T. cruzi* TcI‐JR in C3H/HeN mice inoculated via subcutaneous route.
**Fig. S4**. Survival data.

Supporting info item

Supporting info item

Supporting info item

Supporting info item

Supporting info item
