# Supplementary figures and images for: Host and parasite genetics shape a link between Trypanosoma cruzi infection dynamics and chronic cardiomyopathy
Source: Cell Microbiol. 2016 May 25;18(10):1429–43. doi: 10.1111/cmi.12584 (PMC5031194; doi:10.1111/cmi.12584)

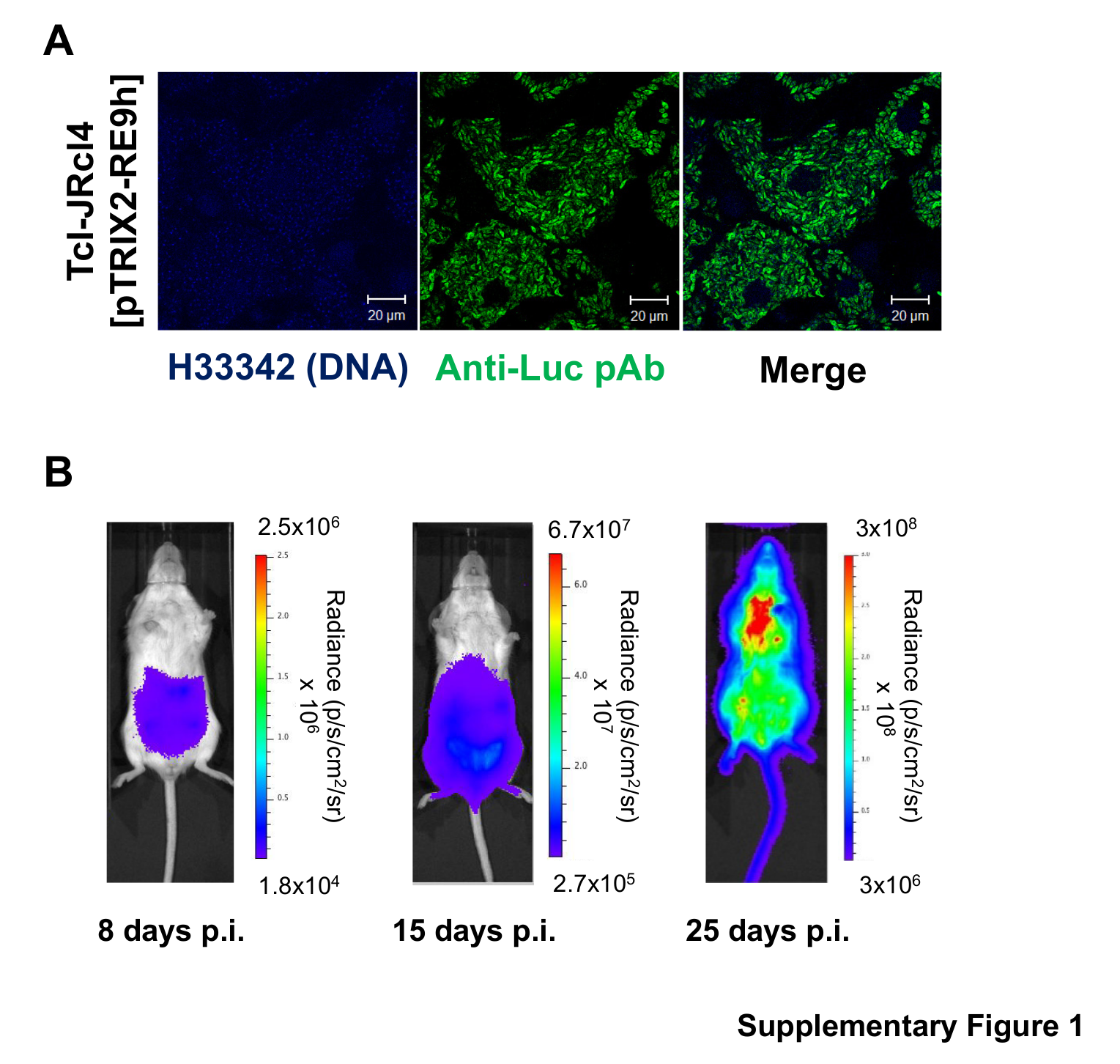

Supplement: Supplementary file 1 — Supporting info item [file CMI-18-1429-s001.tif]

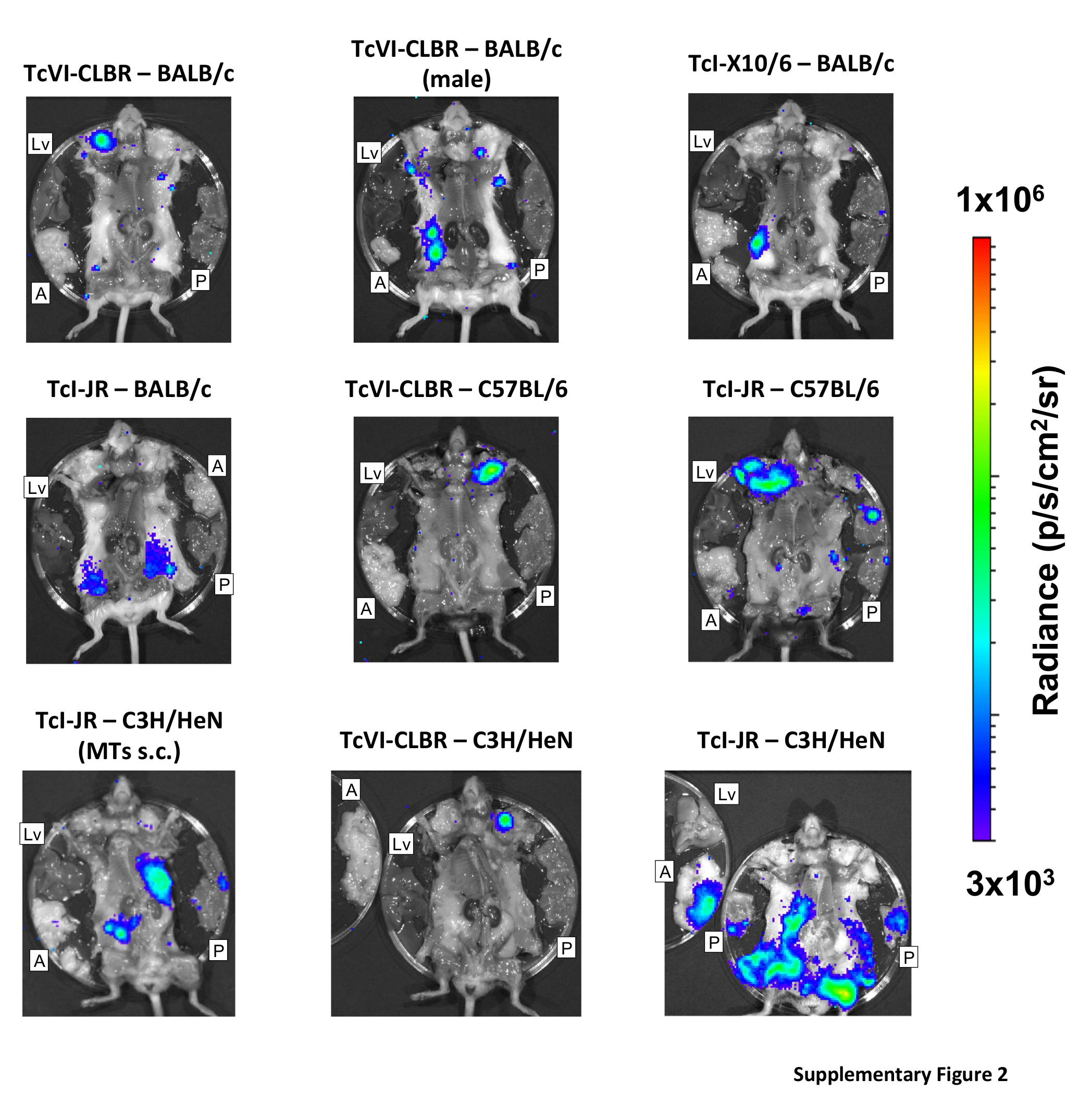

Supplement: Supplementary file 2 — Supporting info item [file CMI-18-1429-s002.tif]

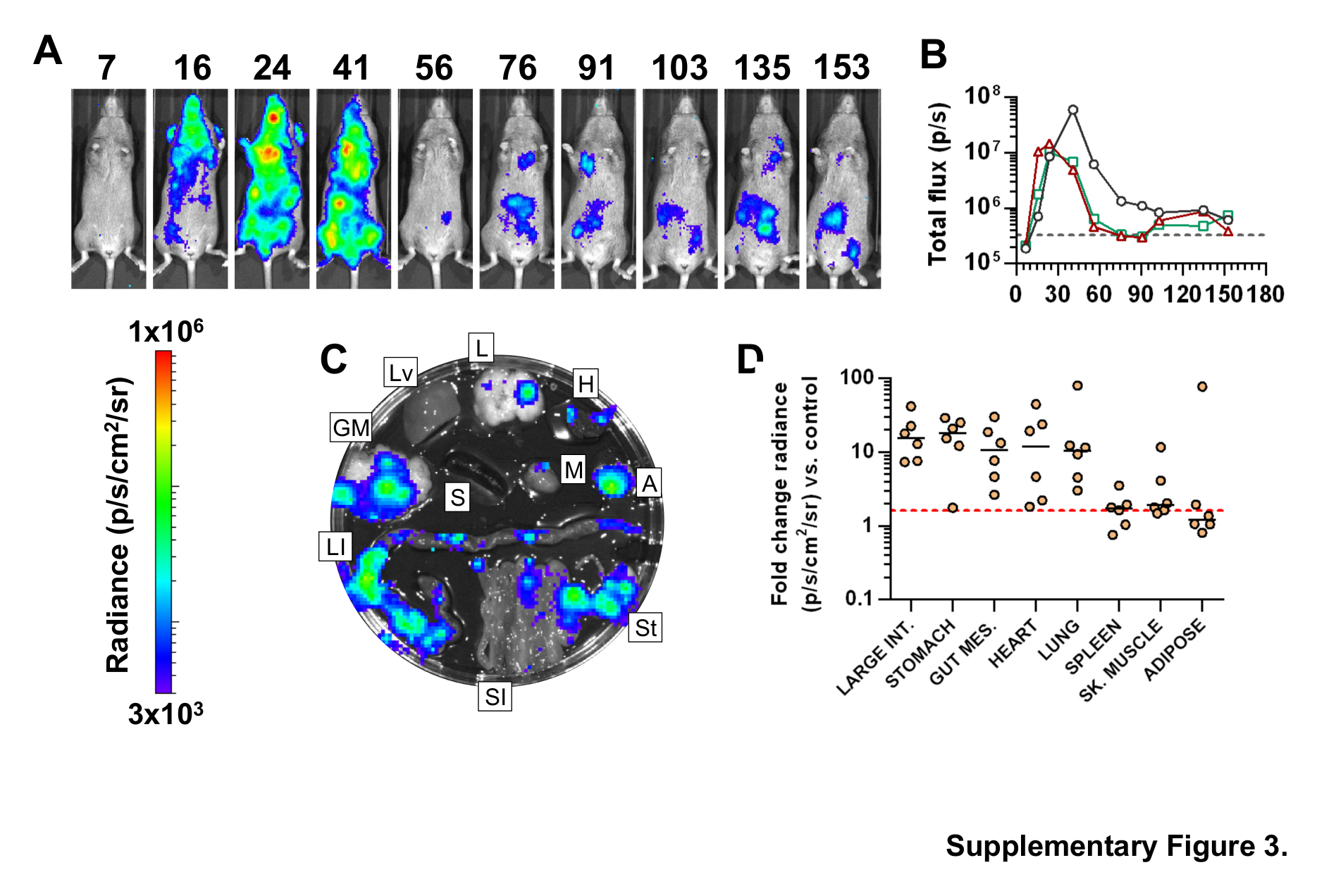

Supplement: Supplementary file 3 — Supporting info item [file CMI-18-1429-s003.tif]

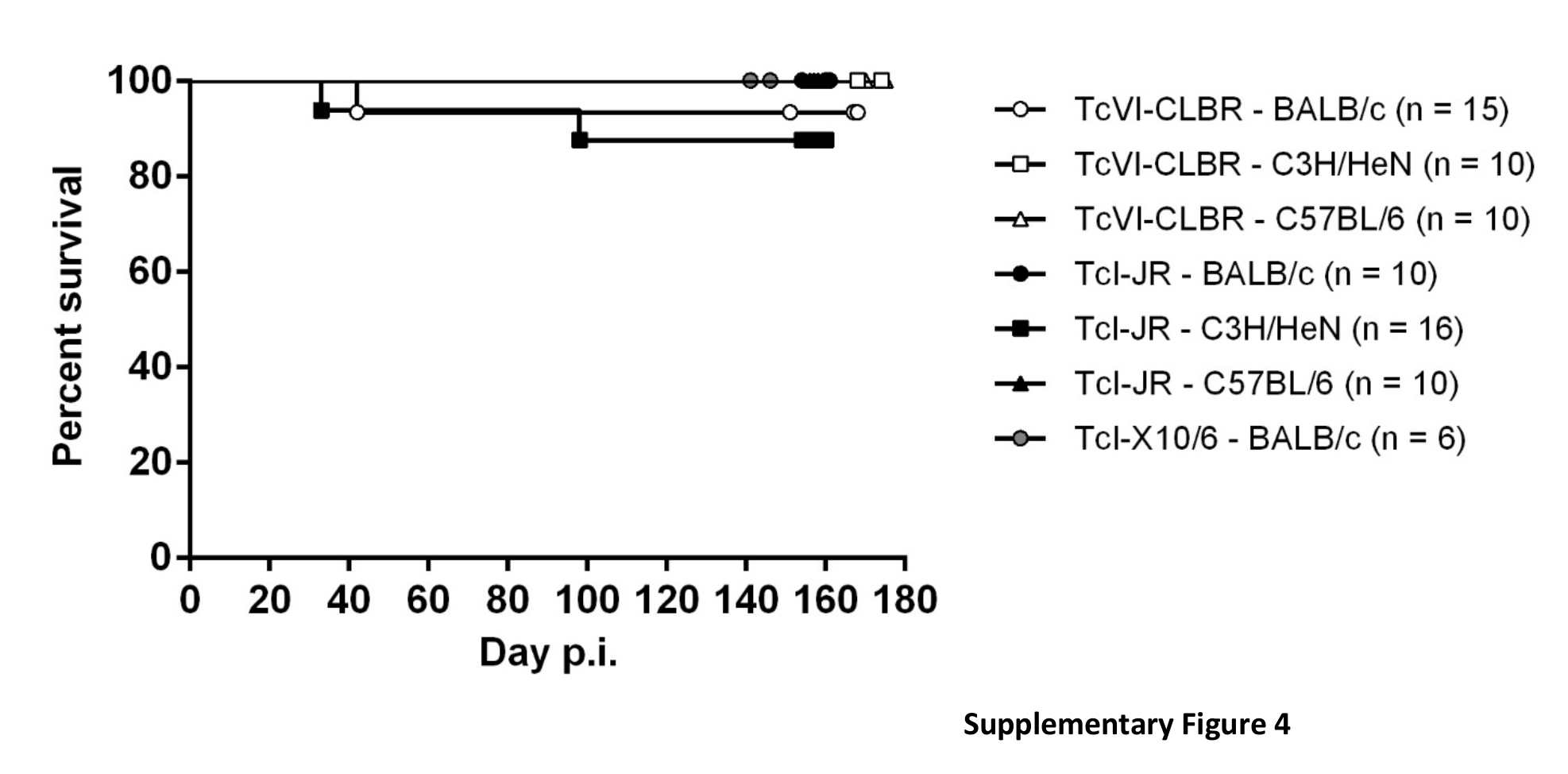

Supplement: Supplementary file 4 — Supporting info item [file CMI-18-1429-s004.tif]
