# Supplementary material for: Host and parasite genetics shape a link between Trypanosoma cruzi infection dynamics and chronic cardiomyopathy
Source: Cell Microbiol. 2016 May 25;18(10):1429–43. doi: 10.1111/cmi.12584 (PMC5031194; doi:10.1111/cmi.12584)
Supplement: Supplementary file 5 — Supporting info item [file CMI-18-1429-s005.pdf]

Table S1. Summary of tissue-specific chronic infection frequencies across *T. cruzi* – mouse strain combinations

Study	Experiment Parameters				Percent of animals bioluminscence positive									
	T. cruzi strain	Mouse (F/M)	Inoculum	n[a]	Heart	Lung	Spleen	SKM	VA	GM	LI	ST	LI or ST	None
This study	TcVI-CLBR	Balb/c (F)	$1x10^3$ BTs i.p.	9	22	22	22	33	22	33	89	89	100	0
This study	TcVI-CLBR	Balb/c (M)	$1x10^3$ BTs i.p.	5	20	40	20	80	20	80	100	80	100	0
Lewis et al 2014	TcVI-CLBR	Balb/c (F)	$1x10^3$ BTs i.p.	8[b]	0	20	13	25	0	25	88	63	100	0
Lewis et al 2014	TcVI-CLBR	Balb/c (F)	$1x10^3$ BTs s.c.	6	0	0	0	0	0	0	83	17	83	17
Both	TcVI-CLBR	Balb/c (F)	$1x10^3$ BTs i.p.	28[c]	11	20	14	32	12	32	89	64	96	4
This study	TcI-X10/6	Balb/c (F)	$2x10^5$ TCTs i.p.	6	17	0	17	67	33	100	100	100	100	0
This study	TcI-JR	Balb/c (F)	$1x10^3$ BTs i.p.	10	50	60	0	60	10	50	80	100	100	0
This study	TcVI-CLBR	C57BL/6 (F)	$1x10^3$ BTs i.p.	10	0	40	40	0	10	80	100	80	100	0
This study	TcI-JR	C57BL/6 (F)	$1x10^3$ BTs i.p.	10	40	30	0	70	10	70	100	90	100	0
This study	TcVI-CLBR	C3H/HeN (F)	$1x10^3$ BTs i.p.	10	10	60	20	30	10	50	80	60	90	0
This study	TcI-JR	C3H/HeN (F)	$1x10^3$ BTs i.p.	8	88	88	25	88	75	100	100	100	100	0
This study	TcI-JR	C3H/HeN (F)	$1x10^3$ MTs s.c.	6	100	100	67	100	50	100	100	100	100	0
All TcI				40	58	55	18	75	33	80	95	98	100	0
All TcVI				48	8	33	21	25	11	46	90	67	96	2
All Models				88	31	44	19	48	21	61	92	81	98	1

M, male; F, female; SKM, skeletal muscle; VA, visceral adipose; GM, gut mesenteries; LI, large intestine; ST, stomach; BT, blood trypomastigotes; MT, metacyclic trypomastigote

[a] survived to end-point

[b] Lewis *et al*. (2014) Cellular Microbiology 16: 1285-1300

[c] lung and adipose n = 5

[d] lung and adipose n = 25
